# COVID-19–Related Fatalities and Intensive-Care-Unit Admissions by Age Groups in Europe: A Meta-Analysis

**DOI:** 10.3389/fmed.2020.560685

**Published:** 2021-01-14

**Authors:** Jérémie F. Cohen, Daniël A. Korevaar, Soraya Matczak, Martin Chalumeau, Slimane Allali, Julie Toubiana

**Affiliations:** ^1^Université de Paris, Centre of Research in Epidemiology and Statistics-CRESS, INSERM, Paris, France; ^2^Department of General Pediatrics and Pediatric Infectious Diseases, AP-HP, Necker Hospital for Sick Children, Université de Paris, Paris, France; ^3^Department of Respiratory Medicine, Amsterdam University Medical Center, University of Amsterdam, Amsterdam, Netherlands

**Keywords:** coronavirus, SARS-CoV-2, COVID-19, mortality, epidemiology, age, meta-analysis, intensive care unit

## Abstract

**Objectives:** Precise international estimates of the age breakdown of COVID-19–related deaths and intensive-care-unit (ICU) admissions are lacking. We evaluated the distribution of COVID-19–related fatalities and ICU admissions by age groups in Europe.

**Materials and methods:** On April 6, 2020, we systematically reviewed official COVID-19–related data from 32 European countries. We included countries that provided data regarding more than 10 COVID-19–related deaths stratified by age according to pre-specified age groups (i.e., <40, 40–69, ≥70 years). We used random-effects meta-analysis to summarize the data.

**Results:** Thirteen European countries were included in the review, for a total of 31,864 COVID-19–related deaths (range: 27–14,381 per country). In the main meta-analysis (including data from Germany, Hungary, Italy, The Netherlands, Portugal, Spain, Switzerland; 21,522 COVID-19–related fatalities), the summary proportions of individuals <40, 40–69, and ≥70 years old among all COVID-19–related deaths were 0.1% (0.0–0.2; *I*^2^ 28.6%), 13.0% (10.8–15.4; *I*^2^ 91.5%), and 86.6% (84.2–88.9; *I*^2^ 91.5%), respectively. ICU data were available for four countries (France, Greece, Spain, Sweden). The summary proportions of individuals around <40–50, around 40–69, and around ≥60–70 years old among all COVID-19–related ICU admissions were 5.4% (3.4–7.8; *I*^2^ 89.0%), 52.6% (41.8–63.3; *I*^2^ 98.1%), and 41.8% (32.0–51.9; *I*^2^ 99%), respectively.

**Conclusions:** People under 40 years old represent a small fraction of most severe COVID-19 cases in Europe. These results may help health authorities respond to public concerns and guide future physical distancing and mitigation strategies. Specific measures to protect older people should be considered.

## Introduction

As of April 6, 2020, more than 1,000,000 confirmed cases and 65,000 deaths due to coronavirus disease 2019 (COVID-19) have been reported globally (1). Data from China and Italy have indicated that older adults are at higher risk of dying from COVID-19 than are younger people (2–4). However, most studies have emphasized the case-fatality rate (4–6) (i.e., the conditional probability of death among classified COVID-19 cases). This indicator is likely to be biased in the early phase of an outbreak, mostly because of preferential testing of people with more severe disease (e.g., hospitalized patients) and delays between the time of death and its official registration (7).

Despite a growing sense that SARS-CoV-2 can result in severe disease regardless of age (8), precise estimates of the age breakdown of COVID-19–related deaths and intensive-care-unit (ICU) admissions are lacking. Here we evaluated the distribution of COVID-19–related fatalities and ICU admissions by age in Europe. Such an analysis is critical to direct any potential relaxation of physical distancing and mitigation measures, which have been highly effective in reducing the number of cases but have severe economic and social consequences (9). We hypothesized that people <40 years old represent a small fraction of most severe COVID-19 cases, as suggested by early reports from the Chinese Centers for Disease Control and Prevention (2).

## Materials and Methods

On April 6, 2020, one of the authors (JFC) systematically reviewed and extracted COVID-19–related mortality data from all 32 European countries participating in the European Center for Disease Prevention and Control surveillance network (i.e., European Union/European Economic Area and the United Kingdom), by collating official reports provided by local Public Health or Ministry of Health websites (Supplementary Table 1). We included countries if they provided data for more than 10 COVID-19–related deaths stratified by age according to pre-specified groups (i.e., <40, 40–69, ≥70 years old). For the included countries, we also extracted data regarding ICU admissions by age groups. A second author (DAK) verified study selection and data extraction, and disagreements were discussed to achieve consensus. We applied no language restrictions.

We used random-effects meta-analysis with Freeman-Tukey double arcsine transformation and exact confidence intervals (CIs) to estimate the proportion of age groups (<40, 40–69, ≥70 years old) among all COVID-19–related fatalities. We excluded participants with missing data for age. For mortality data, we conducted a sensitivity analysis that also included countries reporting data using slightly different age groups, with the following inclusion criteria: (1) the cutoff to define the “younger” age group was within 40–50 years and (2) the “intermediate” age group covered at least 20 years (e.g., United Kingdom, <40, 40–59, ≥60 years). We used the same age criteria to evaluate the distribution of age groups among COVID-related ICU admissions. Heterogeneity was quantified with the *I*^2^ statistic. Statistical analyses involved use of Stata 15/SE (Stata Corp., College Station, TX, USA).

## Results

### Descriptive Data

We identified official reports of COVID-19–related mortality data for all European countries except Cyprus. Eighteen countries (accounting for 1,113 deaths) were excluded because of unusable data: 11 were excluded because of lack of age breakdown of COVID-19 deaths, 5 because of fewer than 10 fatalities, and 2 because of age cutoffs not matching our pre-defined groups (Supplementary Table 1). Thirteen European countries were included in the review, for a total of 31,864 COVID-19–related deaths (range 27–14,381 per country; Table 1) (10–22).

**Table 1:** COVID-19–related fatalities and intensive-care-unit (ICU) admissions by age groups in Europe (as of April 6, 2020).

	Belgium^*^	Finland^**^	France^*^	**Germany**	Greece^***^	**Hungary**	**Italy**	**The Netherlands**	**Portugal**	**Spain**	Sweden^****^	**Switzerland**	UK^**^
Date of report publication	April 6, 2020	April 6, 2020	April 2, 2020	March 29, 2020	April 5, 2020	April 6, 2020	April 5, 2020	April 6, 2020	April 6, 2020	April 3, 2020	April 3, 2020	April 6, 2020	April 6, 2020
Data up to	April 6	March 31	March 29	April 5	Unclear	April 5	April 6	April 5	April 3	March 29	April 6	April 5	April 5
**Number of deaths by age (years)**, ***N*** **(%)**													
<40	9 (0.6)	0 (0.0)	29 (0.8)	1 (0.3)	1 (1.4)	1 (2.6)	42 (0.3)	2 (0.1)	0 (0.0)	13 (0.3)	1 (0.5)	3 (0.5)	43 (0.9)
40–69	112 (6.9)	2 (7.4)	319 (9.1)	46 (11.8)	18 (24.7)	9 (23.7)	2,359 (16.4)	221 (11.8)	41 (13.2)	441 (11.2)	50 (26.3)	61 (10.5)	353 (7.2)
≥70	1,489 (91.2)	25 (92.6)	3,128 (88.8)	341 (87.7)	54 (74.0)	28 (73.7)	11,979 (83.3)	1,643 (88.0)	270 (86.8)	3,050 (77.2)	139 (73.2)	519 (89.0)	4,501 (91.9)
Missing	22 (1.3)	n/a	47 (1.3)	1 (0.3)	n/a	n/a	1 (0.0)	1 (0.1)	n/a	449 (11.4)	n/a	n/a	n/a
Total	1,632 (100)	27 (100)	3,523 (100)	389 (100)	73 (100)	38 (100)	14,381 (100)	1,867 (100)	311 (100)	3,953 (100)	190 (100)	583 (100)	4,897 (100)
**Number of ICU admissions by age (years)**, ***N*** **(%)**													
<40	n/a	n/a	417 (7.6)	n/a	0 (0.0)	n/a	n/a	n/a	n/a	97 (4.5)	26 (7.7)	n/a	n/a
40–69	n/a	n/a	2,327 (42.4)	n/a	43 (46.2)	n/a	n/a	n/a	n/a	998 (46.0)	215 (63.6)	n/a	n/a
≥70	n/a	n/a	2,744 (50.0)	n/a	50 (53.8)	n/a	n/a	n/a	n/a	664 (30.6)	93 (27.5)	n/a	n/a
Missing	n/a	n/a	0 (0.0)	n/a	0 (0.0)	n/a	n/a	n/a	n/a	430 (19.8)	4 (1.2)	n/a	n/a
Total	n/a	n/a	5,488 (100)	n/a	93 (100)	n/a	n/a	n/a	n/a	2,189 (100)	338 (100)	n/a	n/a

*Age groups*:

* * <45, 45–64, ≥65 years*;

** * <40, 40–59, ≥60 years*;

*** * <40, 40–64, ≥65 years*;

**** * <50, 50–69, ≥70 years. In Greece, intensive care unit admissions are patients under mechanical ventilation (intubated). n/a, not available*.

### COVID-19–Related Fatalities by Age Groups

**Figure 1:**
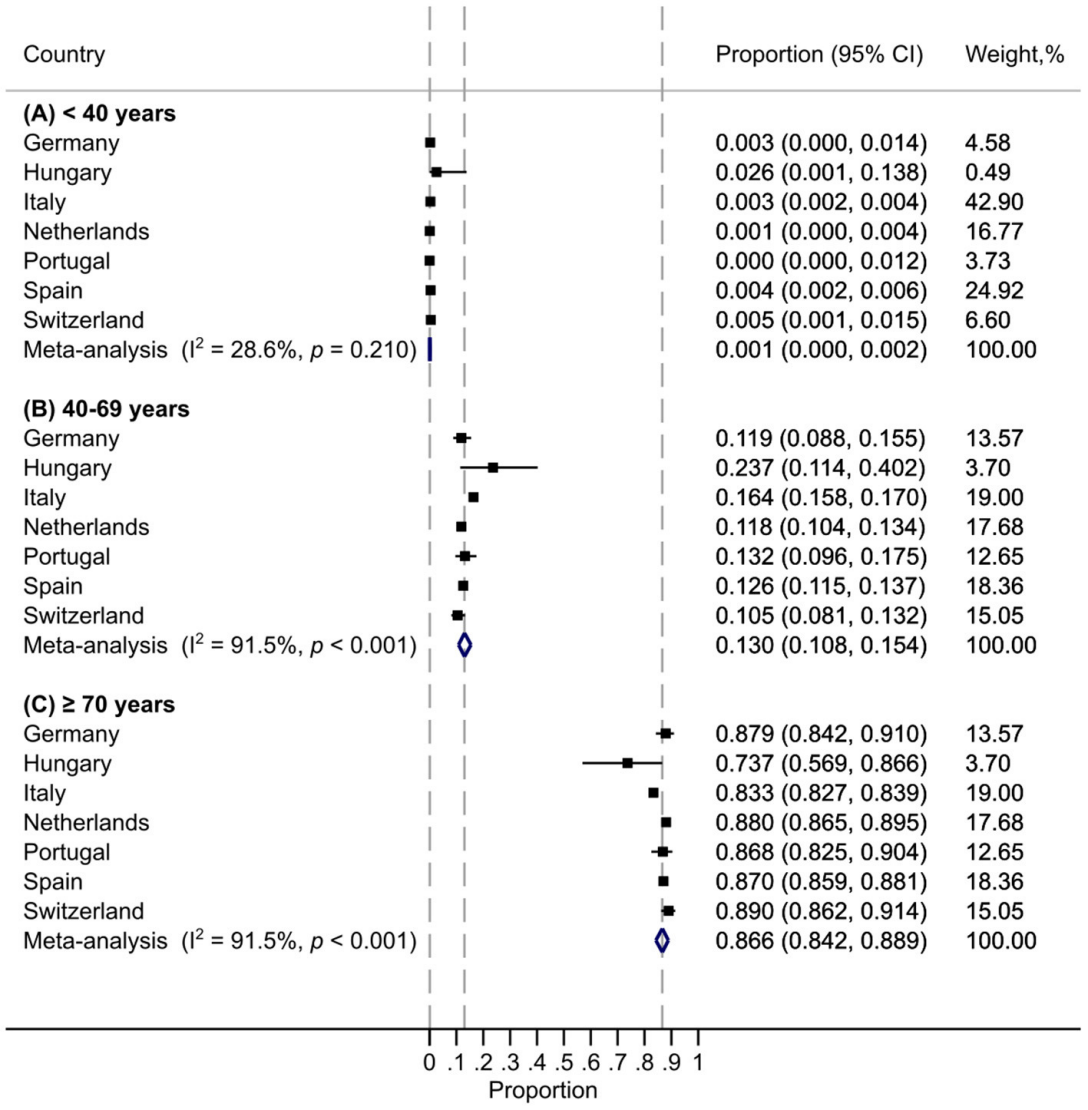
Distribution of age groups for all COVID-19–related deaths in Europe: meta-analysis. Each vertical dashed line is aligned with the meta-analysis summary estimate of the corresponding age group.

In the main meta-analysis (7 countries; 21,522 COVID-19–related fatalities), the summary proportions of individuals <40, 40–69, and ≥70 years old among all COVID-19–related deaths were 0.1% (0.0–0.2; *I*^2^ 28.6%), 13.0% (10.8–15.4; *I*^2^ 91.5%), and 86.6% (84.2–88.9; *I*^2^ 91.5%), respectively (Figure 1). In a sensitivity analysis also including countries that reported slightly different age groups (13 countries; 31,864 COVID-19–related deaths), the summary proportions of individuals around <40–50, around 40–69, and around ≥60–70 years old among all COVID-19–related deaths were 0.2% (0.1–0.4; *I*^2^ 74.6%), 12.7% (10.0–15.7; *I*^2^ 97.4%), and 86.8% (84.0–89.4; *I*^2^ 97.1%), respectively (Supplementary Figure 2).

### COVID-19–Related ICU Admissions by Age Groups

The meta-analysis of ICU admissions included data from 4 countries (8,088 COVID-19–related ICU admissions). The summary proportions of individuals around <40–50, around 40–69, and around ≥60–70 years old among all COVID-19–related ICU admissions were 5.4% (3.4–7.8; *I*^2^ 89.0%), 52.6% (41.8–63.3; *I*^2^ 98.1%), and 41.8% (32.0–51.9; *I*^2^ 99%), respectively (Table 1 and Supplementary Figure 3).

## Discussion

This report describes the current distribution of COVID-19–related deaths and ICU admissions in Europe across age groups, using official reports from 13 countries. These data represent about half of the total of COVID-19–related deaths reported worldwide as of April 6, 2020 (1). Individuals <40 years old represented about 0.1 and 5% of COVID-19–related deaths and ICU admissions, respectively, whereas those >70 years old represented about 85 and 40%, respectively.

The distribution of COVID-19–related deaths by age in Europe differs from what was observed in the early phase of the pandemic in mainland China (as of February 11, 2020: <40, 40–69, ≥70 years old, representing 2.5, 46.6, and 50.8% of 1,023 COVID-19–related deaths, respectively) (4) and what is currently observed in the United States (as of April 7: <45, 45–64, ≥65 years old, representing 3.3, 17.6, and 79.0% of 2,214 COVID-19–related deaths, respectively) (23). This observation could reflect different patterns of patient characteristics, underlying risk factors, and management across settings as well as variability in the organization of healthcare and identification of causes of death across countries (24, 25). The age structure of each population might also be key: in 2019, Italy had the highest proportion of people aged ≥80 years in Europe (7.2%), whereas in the United States, for example, this proportion was as low as 3.6% in 2010 (26). However, the overall prevalence of obesity (body mass index ≥ 30 kg/m^2^), reported as a risk factor for severe COVID-19, is much higher in the United States than Italy (42.4 vs. 10.5%) (27).

Our study has limitations. First, we could not include all European countries, mainly because official reports on COVID-19–related mortality data by age group were not always available. Second, we could not investigate the potential burden of underlying health conditions and other risk factors such as obesity and diabetes because this information was rarely reported. Third, we investigated COVID-19–related mortality data, but we acknowledge that methods for case ascertainment, case definitions, and SARS-CoV-2 RT-PCR testing strategies varied across countries. Some countries, such as France, provided detailed information for only in-hospital fatalities at the time of the study, which does not account for COVID-related deaths that occurred in other settings such as nursing homes (28), for a potential overrepresentation of younger patients. The definition of ICU cases also varied across countries, and Greece only reported the number of intubated patients.

Physical distancing is currently recommended in many countries for all age groups to slow the spread of COVID-19 and protect older people and, more broadly, the healthcare system. People <40 years old represent a small fraction of the total number of most severe COVID-19 cases in Europe. These results, together with evaluations of the impact of comorbidities and risk factors in the course of COVID-19, may help health authorities respond to public concerns and guide future physical distancing and mitigation strategies. Relaxed physical distancing measures could be considered in people under 40 years of age, but specific measures aiming at protecting older people should be developed (29).

## Data Availability Statement

The original contributions presented in the study are included in the article/Supplementary Materials, further inquiries can be directed to the corresponding author/s.

## Ethics Statement

Ethical review and approval was not required for the study on human participants in accordance with the local legislation and institutional requirements. Written informed consent from the participants' legal guardian/next of kin was not required to participate in this study in accordance with the national legislation and the institutional requirements.

## Author Contributions

JC, MC, and JT: conceptualization. JC and JT: methodology. JC: statistical analysis. JC and DK: data extraction and management. JC and JT: writing—original draft preparation. JC, DK, SM, SA, MC, and JT: writing—review and editing. MC and JT: supervision. All authors contributed to the article and approved the submitted version.

## Conflict of Interest

The authors declare that the research was conducted in the absence of any commercial or financial relationships that could be construed as a potential conflict of interest.
