# Supplementary material for: COVID-19–Related Fatalities and Intensive-Care-Unit Admissions by Age Groups in Europe: A Meta-Analysis
Source: Front Med (Lausanne). 2021 Jan 14;7:560685. doi: 10.3389/fmed.2020.560685 (PMC7840596; doi:10.3389/fmed.2020.560685)

SUPPLEMENTARY MATERIAL

**Disclaimer**

This supplementary material is hosted as supporting information alongside the article “COVID-19-related fatalities and intensive care unit admissions by age groups in Europe: A meta-analysis”, on behalf of the authors, who remain responsible for the accuracy and appropriateness of the content.

**Content**

Supplementary Table S1. Sources of data (all websites accessed April 6, 2020)

Supplementary Figure S2. Distribution of age groups among all COVID-19-related deaths in Europe: Sensitivity analysis including countries with less stringent age groups

Supplementary Figure S3. Distribution of age groups among all COVID-19-related ICU admissions in Europe

Supplementary Table S1. Sources of data (all websites accessed April 6, 2020)

| **Country** | **Website** | **No. of death (Date of report)** | **Included in the review** | **Reason for exclusion** |
| --- | --- | --- | --- | --- |
| Austria | www.sozialministerium.at/Informationen-zum-Coronavirus/Neuartiges-Coronavirus-(2019-nCov).html | 220 (April 6) | No | Breakdown by age not reported |
| Belgium | epidemio.wiv-isp.be/ID/Pages/2019-nCoV_epidemiological_situation.aspx  epistat.wiv-isp.be/covid/ | 1,632 (April 6) | Yes | - |
| Bulgaria | www.mh.government.bg/bg/informaciya-za-grazhdani/potvrdeni-sluchai-na-koronavirus-na-teritoriyata-na-r-blgariya/ | 22 (April 6) | No | Breakdown by age not reported |
| Croatia | www.koronavirus.hr/en | 16 (April 6) | No | Breakdown by age not reported |
| Cyprus | Official data not found  ECDC total | 14 (April 6) | No | No official data |
| Czech Republic | onemocneni-aktualne.mzcr.cz/covid-19 | 72 (April 6) | No | Inappropriate age groups (< 50, 50-59, ≥ 60) |
| Denmark | www.sst.dk/da/corona/tal-og-overvaagning | 187 (April 6) | No | Inappropriate age groups (0-59, 60-69, 70-79, 80-89, ≥90) |
| Estonia | www.terviseamet.ee/et/uuskoroonaviirus | 19 (April 6) | No | Breakdown by age not reported |
| Finland | thl.fi/fi/web/infektiotaudit-ja-rokotukset/ajankohtaista/ajankohtaista-koronaviruksesta-covid-19/tilannekatsaus-koronaviruksesta | 27 (April 6) | Yes | - |
| France | www.santepubliquefrance.fr/maladies-et-traumatismes/maladies-et-infections-respiratoires/infection-a-coronavirus/articles/infection-au-nouveau-coronavirus-sars-cov-2-covid-19-france-et-monde | 3,523 (March 31) | Yes | - |
| Germany | www.rki.de/DE/Content/InfAZ/N/Neuartiges_Coronavirus/nCoV.html  Note: Daily updates of data, but after March 29, age groups reported <60, 60 – 69, 70 – 79, 80 – 89, ≥90 | 389 (March 29) | Yes | - |
| Greece | eody.gov.gr/neos-koronaios-covid-19/ | 73 (April 5) | Yes | - |
| Hungary | koronavirus.gov.hu/ | 38 (April 6) | Yes | - |
| Iceland | www.covid.is/data | 4 (April 6) | No | < 10 fatalities |
| Ireland | www.gov.ie/en/publication/967de6-an-analysis-of-the-4443-cases-in-ireland-as-of-friday-3-april-2020/ | 151 (April 3) | No | Breakdown by age not reported |
| Italy | www.epicentro.iss.it/coronavirus/ | 14,381 (April 6) | Yes | - |
| Latvia | arkartassituacija.gov.lv/ | 1 (April 6) | No | < 10 fatalities |
| Liechtenstein | www.regierung.li/coronavirus | 1 (April 4) | No | < 10 fatalities |
| Lithuania | lietuva.lt/en/situation-reports/ | 11 (April 5) | No | Breakdown by age not reported |
| Luxembourg | coronavirus.gouvernement.lu/en.html | 36 (April 5) | No | Breakdown by age not reported |
| Malta | deputyprimeminister.gov.mt/en/health-promotion/Pages/Novel-coronavirus.aspx | 0 (April 6) | No | < 10 fatalities |
| Netherlands | www.rivm.nl/actuele-informatie-over-coronavirus/data | 1867 (April 6) | Yes | - |
| Norway | www.fhi.no/en/id/infectious-diseases/coronavirus/daily-reports/daily-reports-COVID19/ | 59 (April 6) | No | Breakdown by age not reported |
| Poland | www.gov.pl/web/koronawirus/wykaz-zarazen-koronawirusem-sars-cov-2 | 102 (April 6) | No | Breakdown by age not reported |
| Portugal | covid19.min-saude.pt/relatorio-de-situacao/ | 311 (April 5) | Yes | - |
| Romania | datelazi.ro/  www.cnscbt.ro/index.php  stirioficiale.ro/informatii/buletin-de-presa-6-aprilie-2020-ora-13-00 | 168 (April 6) | No | Breakdown by age not reported |
| Slovakia | covid-19.nczisk.sk/sk | 0 (April 6) | No | < 10 fatalities |
| Slovenia | www.gov.si/teme/koronavirus/  www.nijz.si/sl/dnevno-spremljanje-okuzb-s-sars-cov-2-covid-19 | 30 (April 6) | No | Breakdown by age not reported |
| Spain | www.isciii.es/QueHacemos/Servicios/VigilanciaSaludPublicaRENAVE/EnfermedadesTransmisibles/Paginas/InformesCOVID-19.aspx | 3,953 (April 6) | Yes | - |
| Sweden | www.folkhalsomyndigheten.se/folkhalsorapportering-statistik/statistik-a-o/sjukdomsstatistik/covid-19-veckorapporter/senaste-covidrapporten/ | 190 (April 6) | Yes | - |
| Switzerland | www.bag.admin.ch/bag/fr/home/krankheiten/ausbrueche-epidemien-pandemien/aktuelle-ausbrueche-epidemien/novel-cov/situation-schweiz-und-international.html#1164290551 | 583 (April 6) | Yes | - |
| UK | www.england.nhs.uk/statistics/statistical-work-areas/covid-19-daily-deaths/ | 4,897 (April 6) | Yes | - |

Supplementary Figure S2. Distribution of age groups among all COVID-19-related deaths in Europe: Sensitivity analysis including countries with less stringent age groups


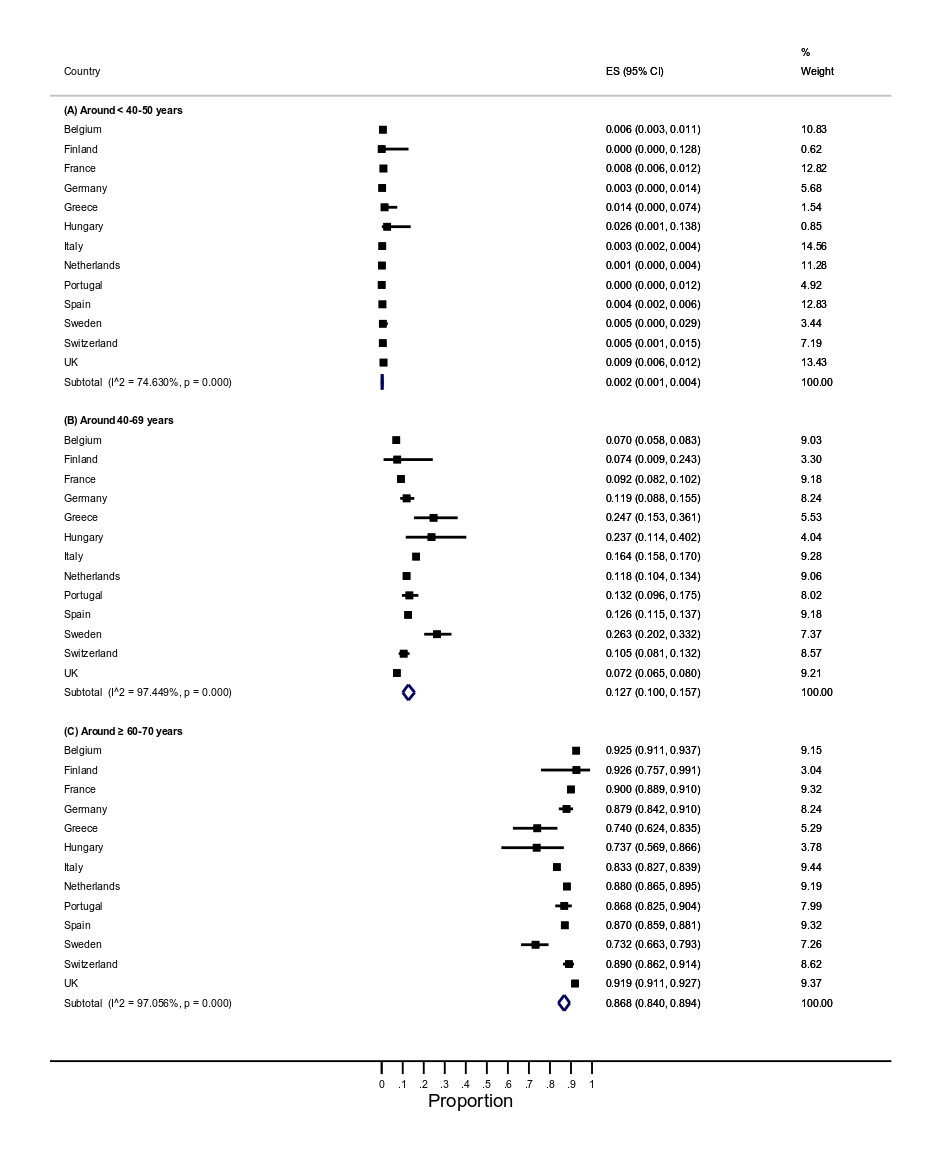


Supplementary Figure S3. Distribution of age groups among all COVID-19-related ICU admissions in Europe


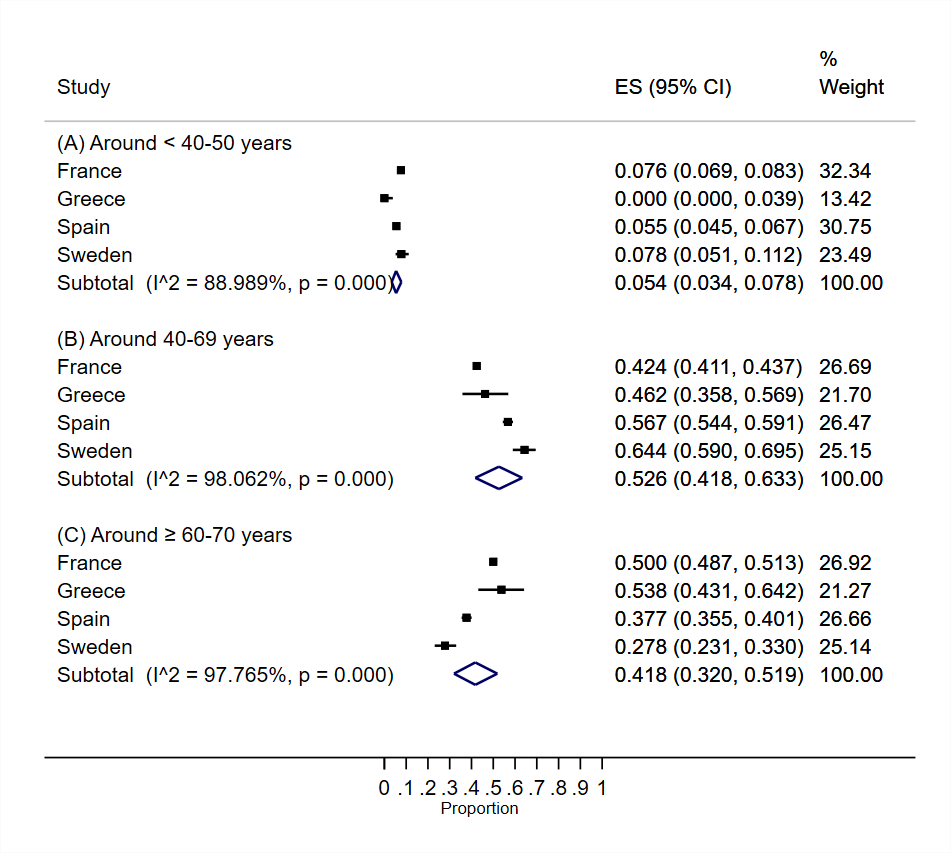

Supplement: Supplementary file 1 [file Table_1.DOCX]
